# Elevated Plasma Big Endothelin-1 at Admission Is Associated With Poor Short-Term Outcomes in Patients With Acute Decompensated Heart Failure

**DOI:** 10.3389/fcvm.2021.629268

**Published:** 2021-03-11

**Authors:** Ran Mo, Yan-min Yang, Li-tian Yu, Hui-qiong Tan, Jun Zhu

**Affiliations:** State Key Laboratory of Cardiovascular Disease, Emergency and Intensive Care Center, National Center for Cardiovascular Diseases, Fuwai Hospital, Chinese Academy of Medical Sciences and Peking Union Medical College, Beijing, China

**Keywords:** acute decompensated heart failure, big endothelin-1, NT- pro B-type natriuretic peptide, short-term prognosis, intensive care

## Abstract

**Objective:** We aimed to evaluate the association between plasma big endothelin-1 (ET-1) at admission and short-term outcomes in acute decompensated heart failure (ADHF) patients.

**Methods:** In this single-center, retrospective study, a total of 746 ADHF patients were enrolled and divided into three groups according to baseline plasma big ET-1 levels: tertile 1 (<0.43 pmol/L, *n* = 250), tertile 2 (between 0.43 and 0.97 pmol/L, *n* = 252), and tertile 3 (>0.97 pmol/L, *n* = 244). The primary outcomes were all-cause death, cardiac arrest, or utilization of mechanical support devices during hospitalization. Logistic regression analysis and net reclassification improvement approach were applied to assess the predictive power of big ET-1 on short-term outcomes.

**Results:** During hospitalization, 92 (12.3%) adverse events occurred. Etiology, arterial pH, lactic acid, total bilirubin, serum creatine, serum uric acid, presence of atrial fibrillation and N-terminal pro-B-type natriuretic peptide (NT-proBNP) levels were positively correlated with plasma big ET-1 level, whereas systolic blood pressure, serum sodium, hemoglobin, albumin, and estimated glomerular filtration rate were negatively correlated. In multivariate logistic regression, tertile 3 compared with tertile 1 had a 3.68-fold increased risk of adverse outcomes [odds ratio (OR) = 3.681, 95% confidence interval (CI) 1.410–9.606, *p* = 0.008]. However, such adverse effect did not exist between tertile 2 and tertile 1 (OR = 0.953, 95% CI 0.314–2.986, *p* = 0.932). As a continuous variable, big ET-1 level was significantly associated with primary outcome (OR = 1.756, 95% CI 1.413–2.183, *p* < 0.001). The C statistic of baseline big ET-1 was 0.66 (95% CI 0.601–0.720, *p* < 0.001). Net reclassification index (NRI) analysis showed that big ET-1 provided additional predictive power when combining it to NT-proBNP (NRI = 0.593, *p* < 0.001).

**Conclusion:** Elevated baseline big ET-1 is an independent predictor of short-term adverse events in ADHF patients and may provide valuable information for risk stratification.

## Introduction

Acute decompensated heart failure (ADHF) is one of the most common and life-threatening diseases in the clinic, causing a high mortality and readmission rate (1). Recent data suggest that the in-hospital mortality for ADHF patients is nearly 3%, whereas the rehospitalization rate exceeds 50% within 6 months (2–4). In addition, the incidence of acute heart failure (AHF) syndrome has increased markedly in the last decades parallel to the aging of the population, a fact that caused a significant disease and economic burden. Therefore, it is essential to identify high-risk ADHF patients at admission and reasonably allocating limited hospital resources to deal with the most urgent situations (5).

Clinical and biochemical determinants of ADHF prognosis have been extensively studied, including age, blood lactate, serum creatinine, heart rate, liver function, serum sodium, and cardiovascular comorbidities. Among them, N-terminal pro-B-type natriuretic peptide (NT-proBNP) is the most widely used laboratory index to evaluate the severity and prognosis of ADHF. In the recent three decades, the endothelin system has been found to play a central role in the pathophysiology of many cardiovascular diseases, including hypertension (6), atherosclerosis (7), coronary artery disease (CAD) (8), and pulmonary arterial hypertension (PAH) (9). Endothelin-1 (ET-1) is the most potent vasoconstrictor, which is produced from the prepropeptide, big ET-1, by endothelin converting enzymes. With a longer half-life time in the peripheral circulation than ET-1, big ET-1 is now considered more suitable for clinical surveillance and prognostic evaluation. However, in the setting of ADHF, the prognostic role of baseline plasma big ET-1 still remains unclear. Thus, the present study aimed to investigate whether elevated plasma big ET-1 at admission is associated with worse short-term outcomes in patients with ADHF and compare its prognostic ability with NT-proBNP. We hypothesized that big ET-1 was a potential factor for improving the risk stratification of ADHF.

## Materials and Methods

### Study Population

This is a retrospective observational study. From January 2014 to December 2018, a total of 746 patients diagnosed with ADHF who were admitted to the intensive care unit (ICU) from the emergency department (ED) at Fuwai Hospital were enrolled in the present study. All participants met the most recent European guidelines for the diagnosis of AHF (10), and ADHF was defined as exacerbation of chronic heart failure (CHF) with worsening symptoms needed intensive care. Additional inclusion criteria for the analysis were: (1) age ≥18 years, (2) ADHF as the first-listed diagnosis, and (3) available baseline big ET-1 level. The following criteria excluded patients from the study: known diagnosis of malignancy, ST-segment elevation myocardial infarction (STEMI), or non-ST-segment elevation myocardial infarction as the leading reason for admission because acute myocardial infarction has a totally different pathogenesis, whereas reperfusion treatment itself plays an important role on both short-term and long-term prognoses. However, patients with combined coronary heart disease (CHD) with CHF who were hospitalized for exacerbation of HF without indications for reperfusion therapy were also included in our study. All clinical data were collected from the electronic medical records. The study was approved by the ethics committee of Fuwai Hospital and was conducted in accordance with the Declaration of Helsinki.

### Data Collection

In patients who entered the study, the detailed baseline data were obtained from their medical records including demographic characteristics, chronic health status, body mass index (BMI), vital signs, and comorbidities. The classification of AHF was in accordance with 2016 European Society of Cardiology (ESC) guidelines (10). The etiology of ADHF was consistent with personal ED records, and the primary diagnosis was adopted when patients had several different pathologies. Vital signs were defined as systolic blood pressure (SBP), diastolic blood pressure (DBP), heart rate (HR), and body temperature measured at the ED. The following laboratory tests were assessed and recorded at admission:

- arterial blood gas: arterial pH, arterial partial pressure of oxygen (PaO_2_), and lactate concentration
- hematology: white blood cell (WBC) count, hemoglobin (Hb) concentration, and hematocrit (HCT)
- Serum electrolytes: sodium, potassium
- Liver and renal functions: plasma albumin, total bilirubin (TBIL), serum uric acid (SUA), and serum creatinine (Scr), and the Chinese version of the Modification of Diet in Renal Disease (MDRD) equation was applied to calculate the participants' estimated glomerular filtration rate (eGFR) (11).
- High sensitivity troponin I (hs-TNI) and NT-proBNP.

The presence of atrial fibrillation (AF) and bundle branch block (BBB) was evaluated by 12-lead electrocardiography, and left ventricular ejection fraction (LVEF) as well as estimated pulmonary arterial systolic pressure (PASP) were measured by experienced physicians using echocardiography. The LVEF was calculated by the modified Simpson's biplane rule.

### Study Grouping and Outcomes

Venous blood samples were drawn from all patients immediately on admission according to standard venous blood specimen collection procedures. To measure the concentration of plasma big ET-1, the medical examination center utilized a highly sensitive and specified Big ET-1 ELISA Kit (BI2008 2H; Biomedica, Wien, Austria). The normal range was <0.25 pmol/L. After a brief analysis of selected patients, we divided them into three groups according to the value of plasma big ET-1: tertile 1 (<0.43 pmol/L, *n* = 250), tertile 2 (between 0.43 and 0.97 pmol/L, *n* = 252), and tertile 3 (>0.97 pmol/L, *n* = 244).

The primary outcome of interest was a composite endpoint defined as: (1) in-hospital death, (2) cardiac arrest occurring during hospitalization, and (3) utilization of mechanical support devices including extracorporeal membrane oxygenation (ECMO). The secondary outcome was all-cause mortality or listed for heart transplantation (HTx).

### Statistical Analysis

For baseline characteristic information, categorical variables were expressed as frequencies (percentages), and continuous variables were expressed as means ± standard deviations (SD) or medians with quartiles if they were not in the normal distribution. Normality was calculated using the Shapiro–Wilk W-test. A log-data transformation was applied to fit skewed distributions to normal distributions, such as eGFR, hs-TNI, and NT-proBNP. Variance analysis was adopted to compare baseline continuous variables and Pearson's chi-squared test or Fisher's exact test for categorical variables among tertile 1, tertile 2, and tertile 3. Factors related to plasma big ET-1 level were assessed by Spearman correlation analysis. Univariate logistic regression was used to evaluate the predictive power for short-term outcomes of big ET-1 and other clinical parameters, whereas odds ratios (ORs) and their 95% confidence intervals (95% CIs) were displayed. Then, based on univariate analysis, several statistically significant predictors were included in multivariate logistic regression model with a forward stepwise selection algorithm. Subsequently, in order to test the predictive power of big ET-1, we performed receiver-operating characteristic (ROC) curve and used the optimal cut-off value of baseline NT-proBNP to recategorize the patients (group 1: NT-proBNP <14,873 pg/ml, *n* = 654; group 2: NT-proBNP ≥ 14,873 g/ml, *n* = 92). The area under the curve (AUC), net reclassification index (NRI), and integrated discrimination improvement (IDI) were calculated to further compare the prediction performance of these two parameters.

The software package SPSS version 25.0 (IBM Corporation, New York, NY, USA) and SAS 9.4 (SAS Institute, Cary, NC, USA) were utilized for statistical analysis. All statistical tests were two-tailed, with a *p* < 0.05 considered statistically significant. Graphs were generated using the software GraphPad Prism 8.0.

## Results

### Baseline Characteristics

The baseline characteristics of total participants and different groups were shown in Table 1. The mean age of the study population was 58.3 ± 16.6 years, and female only accounted for 28.3%. Age and sex distribution showed no statistical difference in the three groups. In total, 707 (94.8%) patients were categorized as congestive AHF. Two hundred nine (28%) participants had diabetes mellitus, and 34.3% had AF on the electrocardiogram. The top three causes for ADHF were ischemic heart disease (42.4%), cardiomyopathy (32.8%), and valvular disease (13.4%). Patients in tertile 2 and tertile 3 had lower SBP (*p* = 0.016), faster HR (*p* = 0.002), and apparently more manifestations of AF (*p* < 0.001) as well as pulmonary hypertension (*p* < 0.001). For blood laboratory test, those who had elevated big ET-1 were more likely with higher levels of arterial pH (*p* = 0.018), lactic acid (*p* = 0.008), TBIL (*p* < 0.001), SUA (*p* < 0.001), Scr (*p* < 0.001), hs-TNI (*p* < 0.001), and NT-proBNP (*p* < 0.001). In the meantime, they had significant lower levels of serum sodium (*p* < 0.001), Hb (*p* < 0.001), albumin (*p* < 0.001), and eGFR (*p* < 0.001).

**Table 1:** Baseline characteristics based on big ET-1 tertiles.

**Variables**	**Total (*n =* 746)**	**Tertile 1 (*n =* 250)**	**Tertile 2 (*n =* 252)**	**Tertile 3 (*n =* 244)**	***p-*value**
**Demographics**					
Age (years)	58.3 ± 16.6	58.1 ± 15.8	59.9 ± 16.5	56.9 ± 17.4	0.112
Sex (female, %)	211 (28.3%)	77 (30.8%)	69 (27.4%)	65 (26.6%)	0.547
ADHF type (congestion, %)	707 (94.8)	241 (96.4)	240 (95.2)	226 (92.6)	0.155
**Etiology of HF (*****n*****, %)**					**0.005**
Ischemic heart disease	316 (42.4)	121 (48.4)	103 (40.9)	92 (37.7)	
Valvular disease	100 (13.4)	30 (12.0)	43 (17.1)	27 (11.1)	
Cardiomyopathy	245 (32.8)	75 (30.0)	70 (27.8)	100 (41.0)	
Immune disorders	25 (3.4)	11 (4.4)	9 (3.6)	5 (2.0)	
Inflammatory damage	16 (2.1)	4 (1.6)	7 (2.8)	5 (2.0)	
Congenital heart disease	27 (3.6)	9 (3.6)	8 (3.2)	10 (4.1)	
Aortic disease	2 (0.3)	0 (0.0)	1 (0.4)	1 77 (0.4)	
Pulmonary heart disease	15 (2.0)	0 (0.0)	11 (4.4)	4 (1.6)	
**Vital signs**					
BMI (kg/m^2^)	24.08 ± 4.51	24.43 ± 4.29	23.68 ± 4.42	24.13 ± 4.79	0.186
**SBP (mmHg)**	**117 ± 42**	**122 ± 66**	**116 ± 21**	**112 ± 21**	**0.016**
DBP (mmHg)	72 ± 14	73 ± 12	73 ± 15	70 ± 13	0.039
Temperature (°C)	36.4 ± 4.0	36.6 ± 6.6	36.4 ± 0.4	36.3 ± 2.2	0.702
**Heart rate (bpm)**	**79 ± 18**	**76 ± 16**	**81 ± 18**	**80 ± 21**	**0.002**
**Comorbidities**					
Smoking (n, %)	385 (51.6)	131 (52.4)	130 (51.6)	124 (50.8)	0.940
Drinking (n, %)	309 (41.4)	107 (42.8)	101 (40.1)	101 (41.4)	0.826
**Diabetes mellitus (*****n*****, %)**	**209 (28)**	**56 (22.4)**	**68 (27.0)**	**85 (34.8)**	**0.008**
**Laboratory test**					
**Arterial pH**	**7.44 ± 0.13**	**7.43 ± 0.20**	**7.44 ± 0.08**	**7.46 ± 0.06**	**0.018**
PaO_2_ (mmHg)	87 ± 24	88 ± 21	86 ± 25	88 ± 26	0.709
**Lactic acid (mmol/L)**	**1.8 ± 1.0**	**1.6 ± 0.8**	**1.7 ± 0.7**	**2.0 ± 1.3**	**0.008**
**Serum sodium (mmol/L)**	**135.66 ± 11.08**	**137.35 ± 9.29**	**136.20 ± 5.15**	**133.37 ± 15.89**	**<0.001**
Serum potassium (mmol/L)	4.23 ± 5.04	4.10 ± 0.49	3.97 ± 0.53	4.63 ± 8.79	0.304
WBC count (×10^9^/L)	7.70 ± 5.62	7.25 ± 2.12	7.53 ± 3.12	8.35 ± 9.03	0.078
**Hemoglobin (g/L)**	**136.3 ± 24.8**	**145.4 ± 22.3**	**134.1 ± 24.5**	**129.5 ± 24.9**	**<0.001**
Hematocrit	0.62 ± 5.44	0.45 ± 0.26	0.41 ± 0.07	1.01 ± 9.51	0.393
**Albumin (g/L)**	**40.0 ± 15.5**	**43.3 ± 24.8**	**38.7 ± 6.05**	**37.9 ± 15.5**	**<0.001**
**Total bilirubin (μmol/L)**	**29.54 ± 21.33**	**21.61 ± 14.72**	**28.07 ± 20.08**	**39.15 ± 24.42**	**<0.001**
**Uric acid (μmol/L)**	**534.2 ± 179.0**	**469.8 ± 142.6**	**509.2 ± 166.3**	**626.1 ± 188.3**	**<0.001**
**Creatinine (μmol/L)**	**110.6 ± 51.3**	**95.4 ± 31.6**	**108.6 ± 50.5**	**128.4 ± 62.1**	**<0.001**
**hs-TNI (μg/L)**	**0.039 (0.020–0.088)**	**0.028 (0.020–0.063)**	**0.039 (0.020–0.077)**	**0.052 (0.022–0.129)**	**<0.001**
**eGFR (ml/min/1.73 m**^**2**^**)**	**63.16 (43.36–89.26)**	**71.93 (52.50–96.93)**	**61.00 (43.46–85.76)**	**58.74 (36.91–82.65)**	**<0.001**
**NT-proBNP (pg/ml)**	**5,124 (2,195.2–11,450.17)**	**2,184.37 (1,091.2–4,620.00)**	**5,226.00 (2,880.15–9,101.80)**	**9,544.40 (4,953.10–14,343.64)**	**<0.001**
**Echocardiography**					
LVEF (%)	37.1 ± 13.0	37.5 ± 11.9	37.2 ± 13.7	36.6 ± 13.4	0.710
**PASP** **>30 mmHg (n, %)**	**195 (26.1)**	**31 (12.4)**	**62 (24.6)**	**102 (41.8)**	**<0.001**
**Electrocardiogram**					
**Atrial fibrillation (n, %)**	**255 (34.3)**	**66 (26.5)**	**84 (33.5)**	**105 (43.2)**	**<0.001**
Bundle branch block (n, %)	178 (24.1)	57 (23.1)	62 (24.8)	59 (24.3)	0.899

*Bold items are statistically significant*.

### Correlations of Variables With Big ET-1

The results of bivariable correlation analysis were listed in Table 2. The following parameters were significantly associated with big ET-1 level on admission: etiology (r = 0.086, *p* = 0.019), SBP (r = −0.088, *p* = 0.016), arterial pH (r = 0.102, *p* = 0.006), lactic acid (r = 0.145, *p* = 0.001), serum sodium (r = −0.112, *p* = 0.002), Hb (r = −0.146, *p* < 0.001), albumin (r = −0.097, *p* = 0.008), TBIL (r = 0.354, *p* < 0.001), Scr (r = 0.246, *p* < 0.001), SUA (r = 0.336, *p* < 0.001), eGFR (r = −0.124, *p* = 0.001), NT-proBNP (r = 0.438, *p* < 0.001) and presence of AF (r= −0.152, *p* < 0.001). Among these factors, log-transformed NT-proBNP had the best correlation.

**Table 2:** Bivariable correlation between big ET-1 and clinical variables.

**Variables**	**r**	***p-*value**
Etiology	0.086	0.019
SBP	−0.088	0.016
Arterial pH	0.102	0.006
Lactic acid	0.145	0.001
Serum sodium	−0.112	0.002
Hemoglobin	−0.146	<0.001
Albumin	−0.097	0.008
Total bilirubin	0.354	<0.001
Creatinine	0.246	<0.001
Uric acid	0.336	<0.001
Lg eGFR	−0.124	0.001
Lg NT-proBNP	0.438	<0.001
Atrial fibrillation	0.152	<0.001

### Outcomes and Multivariate Logistic Regression

**Figure 1:**
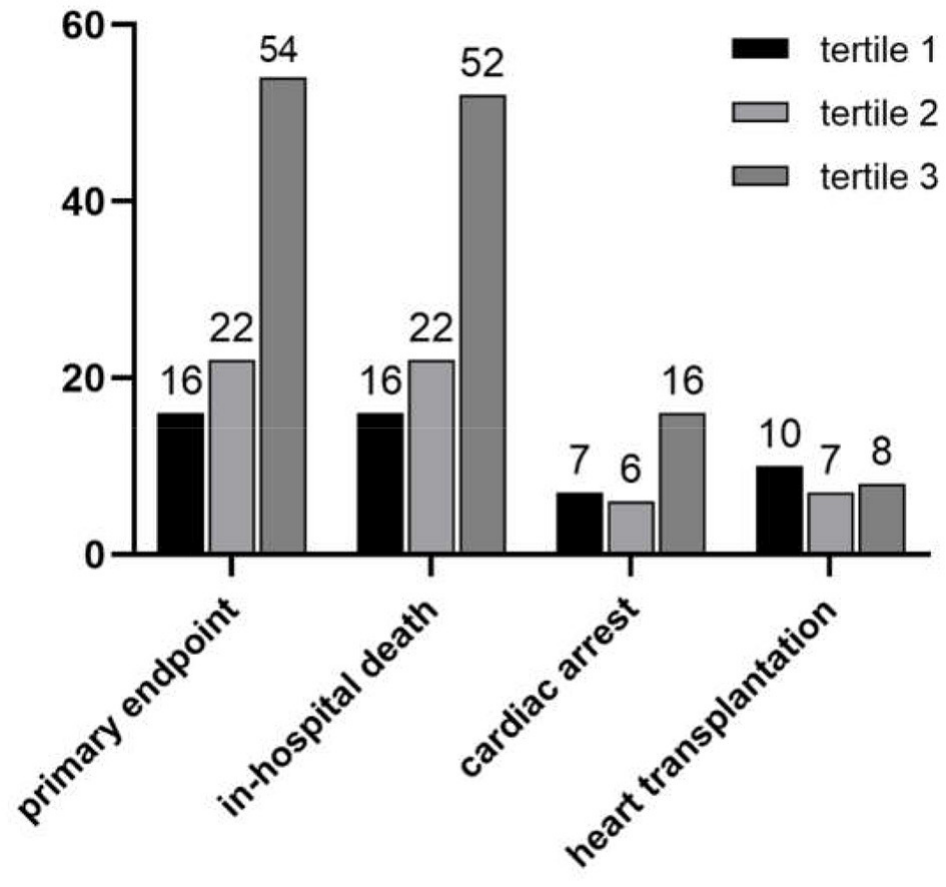
Clinical outcomes based on big ET-1 tertiles.

The clinical outcomes classified by the big ET-1 groups were shown in Figure 1. During hospitalization, 92 (12.3%) primary composite endpoints occurred, of whom 90 (12.1%) patients suffered from in-hospital death, 29 (12.1%) suffered cardiac arrest, and 7 (0.9%) received mechanical support devices therapy. Furthermore, 25 (3.4%) critically-ill patients were listed for HTxs. The tertile 2 and tertile 3 groups had significantly higher rates in both composite primary outcomes (6.4 vs. 8.7 vs. 22.1%, *p* < 0.001) and in-hospital mortality (6.4 vs. 8.7 vs. 21.3%, *p* < 0.0001). However, there was no statistical difference of HTx among the three groups (4.0 vs. 2.8 vs. 3.4%, *p* = 0.773).

Relations between baseline factors and outcomes were shown in Table 3. In the univariate regression, congestion, big ET-1, SBP, DBP, HR, lactic acid, WBC count, albumin, TBIL, SUA, Scr, log-transformed eGFR, and log-transformed NT-proBNP were, respectively, related to the primary composite endpoint. When involving all the parameters into multivariate logistic regression, plasma big ET-1 and WBC count (OR = 1.297, 95% CI 1.186–1.420, *p* < 0.001) were independent risk factors. The highest big ET-1 group compared with the lowest had a 3.68-fold increased risk of adverse outcomes during hospitalization (OR = 3.681, 95% CI 1.410–9.606, *p* = 0.008). Interestingly, such risk did not persist if patients were in tertile 2 compared with those who belonged to tertile 1 (OR = 0.953, 95% CI 0.314–2.986, *p* = 0.932). As a continuous variable, big ET-1 level was also significantly associated with primary outcome (OR = 1.756, 95% CI 1.413–2.183, *p* < 0.001) and in-hospital death (OR = 1.734, 95% CI 1.394–2.158, *p* < 0.001) but not for HTx (OR = 0.931, 95% CI 0.558–1.552, *p* = 0.784).

**Table 3:** Predictor of primary endpoint in uni- and multivariate logistic regression.

**Variables**	**Univariate**		**Multivariate**	
	**OR (95% CI)**	***p***	**OR (95% CI)**	***p***
Big ET-1		<0.001		0.001
Tertile 2	1.399 (0.716–2.732)	0.326	0.953 (0.314–2.986)	0.932
Tertile 3	4.157 (2.305–7.497)	<0.001	3.681 (1.410–9.606)	0.008
Age (years)	0.990 (0.978–1.003)	0.142		
Gender	0.939 (0.575–1.532)	0.801		
Congestion (%)	0.380 (0.179–0.809)	0.012		
Etiology		0.191		
BMI (kg/m^2^)	0.972 (0.922–1.024)	0.288		
SBP (mmHg)	0.983 (0.972–0.994)	0.002		
DBP (mmHg)	0.964 (0.948–0.981)	<0.001		
Temperature (°C)	0.962 (0.883–1.049)	0.381		
HR (bpm)	1.012 (1.001–1.024)	0.036		
Smoking (%)	1.537 (0.94–2.400)	0.059		
Drinking (%)	0.995 (0.638–1.549)	0.981		
DM (%)	0.683 (0.405–1.154)	0.154		
Arterial pH	0.301 (0.067–1.357)	0.118		
PaO_2_ (mmHg)	1.005 (0.996–1.013)	0.276		
Lactic acid (mmol/L)	1.636 (1.293–2.071)	<0.001		
Serum sodium (mmol/L)	0.993 (0.978–1.008)	0.371		
Serum potassium (mmol/L)	0.996 (0.943–1.051)	0.881		
WBC (×10^9^/L)	1.138 (1.069–1.212)	<0.001	1.297 (1.186–1.420)	<0.001
Hb (g/L)	0.995 (0.987–1.004)	0.287		
HCT	0.210 (0.001–4.514)	0.319		
Albumin (g/L)	0.961 (0.931–0.997)	0.031		
TBIL (μmol/L)	1.018 (1.009–1.027)	<0.001		
SUA (μmol/L)	1.003 (1.002–1.004)	<0.001		
Scr (μmol/L)	1.009 (1.006–1.013)	<0.001		
hs-TNI (μg/L)	1.006 (0.980–1.033)	0.637		
Lg eGFR	0.287 (0.108–0.766)	0.013		
Lg NT-proBNP	3.079 (1.706–5.557)	<0.001		
LVEF (%)	1.008 (0.991–1.024)	0.359		
PASP >30 mmHg	1.276 (0.791–2.057)	0.317		
AF	0.898 (0.561–1.438)	0.655		
BBB	0.742 (0.424–1.298)	0.296		

### Predictive Values of Big ET-1 and NT-proBNP

**Figure 2:**
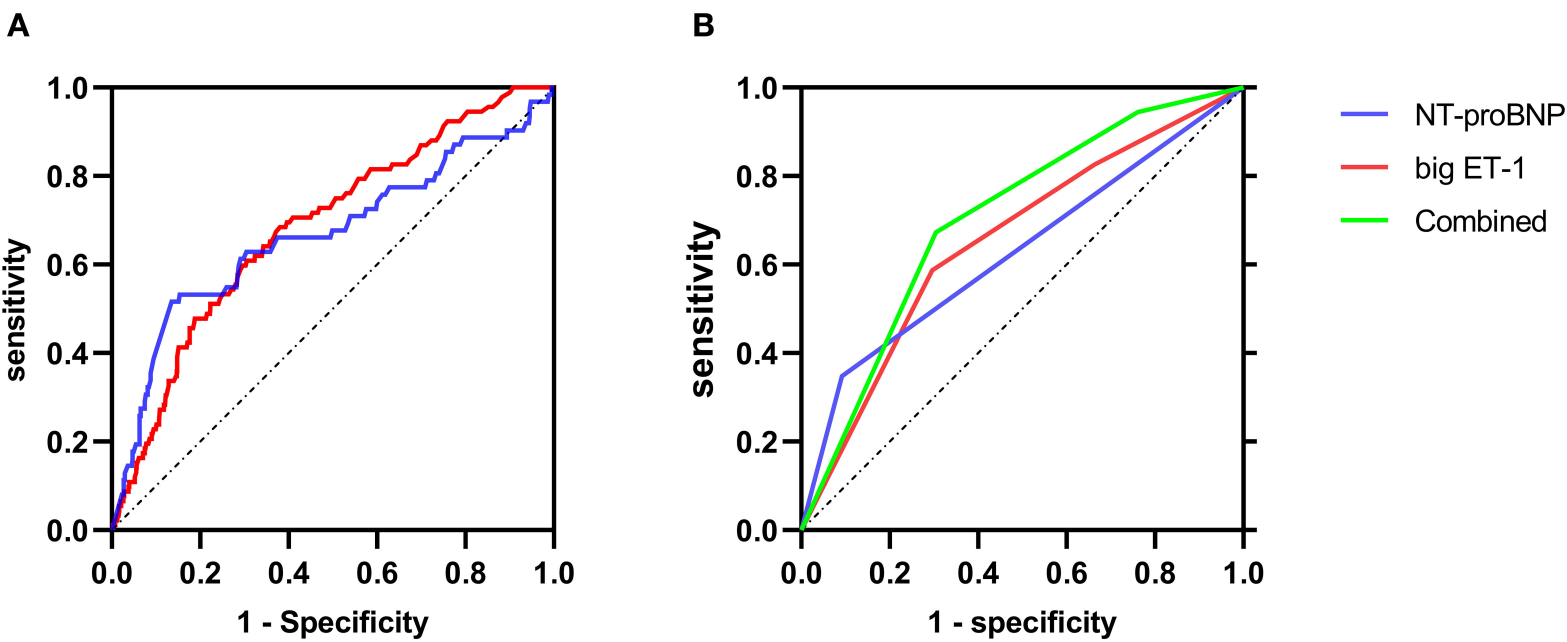
ROC curves of primary endpoint predicted by NT-proBNP and big ET-1. **(A)** The ROC curves when big ET-1 and NT-proBNP were analyzed as continuous variables. **(B)** The ROC curves when big ET-1and NT-proBNP were analyzed as categorical variables, respectively, and the ROC curve for the combination of big ET-1 and NT-proBNP.

ROC curves of big ET-1 and NT-proBNP at admission were shown in Figure 2. As categorical variables, the C statistics were 0.66 for the big ET-1 groups (95% CI 0.601–0.720, *p* < 0.001) and 0.628 for the NT-proBNP groups (95% CI 0.560–0.696, *p* < 0.001) (Figure 2B). When these two parameters were included as continuous variables, the AUC values were 0.685 for big ET-1 level (95% CI 0.628–0.743, *p* < 0.001) and 0.667 for log-transformed NT-proBNP (95% CI 0.584–0.752, *p* < 0.001) (Figure 2A). The NRI and IDI analyses were performed to compare the predictive powers of big ET-1 and NT-proBNP (Supplementary Data 1). Plasma big ET-1 proved to have similar risk stratification as NT-proBNP, the representative indicator for HF patients (NRI = 5.40%, 95% CI −0.16–0.27, *p* = 0.627; IDI = 2.53%, 95% CI −0.002–0.053, *p* = 0.072). When adding big ET-1 levels to baseline NT-proBNP, the C statistics for primary outcomes increased to 0.704 (95% CI 0.644–0.764, *p* < 0.001) and 0.701 for in-hospital death (95% CI 0.640–0.762, *p* < 0.001). A total of 17% of patients were correctly reclassified (NRI = 0.593, 95% CI 0.38–0.81, *p* < 0.001; IDI = 0.0185, 95% CI 0.001–0.0036, *p* = 0.035) (Supplementary Data 2). However, none of the parameters were found to be associated with HTx.

## Discussion

In the present study of Chinese patients in a single heart center ICU setting, we found that plasma big ET-1 was significantly related to the elevated risk of short-term adverse outcomes for ADHF patients who were firstly admitted to the ED. Such predictive power still existed after adjusting other clinical indicators. Moreover, baseline big ET-1 provided additional prognostic information to that yielded only by NT-proBNP. Therefore, big ET-1 as a new and practical biomarker might aid in the identification of ADHF patients at risk for the incidence of in-hospital death, cardiac arrest, or use of mechanical support devices.

Endothelin was first identified in 1988 (12), and the pathophysiological effects of the endothelin system have subsequently been investigated in various conditions including the cardiovascular system (13). ET-1 is recognized as the most potent and long-lasting vasoconstrictor. ET-1 can be synthesized and secreted in many cell types including cardiac myocytes, hepatocytes, kidney epithelial cells, WBCs, macrophages, and endothelial cells (14). Circulating ET-1 produced biological effect *via* binding to two specific receptors, namely, ETA and ETB (15). In heart failure settings, ETA is up-regulated, whereas ETB is down-regulated, causing negative inotropic and proarrhythmic effects. On the one hand, ET-1 stimulates cardiac remodeling by inducing inflammation and renin–angiotensin–aldosterone system. On the other hand, ET-1 also promotes the formation of norepinephrine with vasopressin (13).

A growing number of evidences suggest that elevated plasma big ET-1 level is a significantly independent predictor for CAD (8, 16), cardiomyopathy (17, 18), AF (19), and PAH (20). Several studies aimed at exploring the clinical effect of the endothelin system in heart failure. In CHF, cumulative results have demonstrated that ET system activation is linked to CHF presence, progression, and increased morbidity (21–23). Masson et al. measured baseline plasma big ET-1 levels of 2,359 stable and symptomatic HF patients and found that the circulating concentration of big ET-1 was an independent predictor of long-term all-cause mortality, but its prognostic value was weaker than BNP (24). Perez et al. reported the close associations between continuously measured ET-1 and both in-hospital and long-term outcomes in AHF patients (25). However, existing studies did not clarify the predictive power of plasma big ET-1, as the precursor of ET-1 with a more stable and accurate measurement, for short-term adverse events in critically-ill ADHF patients. In our study, we enrolled 746 consecutive ADHF patients admitted to the ICU and calculated that the AUC for baseline big ET-1 in predicting adverse in-hospital events was 0.66. Interestingly, when bringing big ET-1 and NT-proBNP into multivariable analysis, big ET-1 instead of NT-proBNP was significantly related to short-term outcomes. Besides, through NRI approach, our result indicated that baseline big ET-1 owned similar stratification capacity with NT-proBNP.

Moreover, our study suggested that arterial pH, lactic acid, TBIL, Scr, SUA, and presence of AF and NT-proBNP were positively correlated with plasma big ET-1 level. Conversely, SBP, serum sodium, Hb, albumin, and eGFR were negatively correlated. These findings revealed that the strong endothelin system activation reflected not only cardiac function but also renal and liver functions and personal nutritional status. The important biological functions of this comprehensive indicator in multiple organs were consistent with previous works (13, 24, 26, 27).

Although big ET-1 showed a satisfactory predictive power for the composite endpoint, it cannot accurately predict HTxs. The candidacy for HTx was assessed carefully in Fuwai Hospital. Elderly and frail patients with ADHF who failed optimal medical management and mechanical circulatory support often suffered from malnutrition, immune dysfunction, and multiple organ failure. They were obviously unsuitable for operations. It was understandable that the baseline big ET-1 level was unparallel to the consideration of HTx. Secondly, the selection for HTx was associated with economic conditions, social support, and psychological condition.

Endothelin receptor antagonists (ERAs) have been one of the hot focuses in cardiovascular diseases especially in PAH. Disappointedly, ERAs were found to be less satisfactory as a therapy for HF. The randomized intravenous Tezosentan study failed to show a significant improvement in composite primary endpoint in ADHF with acute coronary syndrome patients, but symptomatic hypotension was more frequent in the treatment group (28). Another randomized double-blind trial demonstrated that Bosentan did not improve clinical long-term outcomes in severe CHF patients but caused early and important fluid retention (29). Big ET-1 assessment may identify a subgroup of ADHF patients who benefit from treatment targeting the endothelin system. More solid evidence is needed in ERAs treating ADHF with high plasma big ET-1 level.

The following were several limitations in the present study. First, our database consisted of a cohort of patients from a single cardiovascular hospital, and the study population included only Chinese patients. The participants evaluated were limited to patients admitted only to the ICU, and ADHF patients who were then admitted to other wards were not enrolled. The results should be carefully interpreted when applied to a larger population. Second, the primary endpoint was in-hospital death or cardiac arrest or clinical application of mechanical support devices. Due to the lack of follow-ups after discharge, the predictive ability of baseline plasma big ET-1 for post-charge prognosis was still unknown. Third, the individual clinical data were collected at admission without taking acute-phase managements into account, such as the widely used inotropic or diuretic drugs for ADHF, which might influence admission laboratory test results. Considering the incompleteness and availability of past medical history in practical ED settings, we lacked information on baseline HF treatments, which might interfere with the big ET-1 prognostic power.

## Data Availability Statement

The raw data supporting the conclusions of this article will be made available by the authors, without undue reservation.

## Ethics Statement

The studies involving human participants were reviewed and approved by ethics committee of Fuwai Hospital. The patients/participants provided their written informed consent to participate in this study.

## Author Contributions

RM collected the clinical data, did the analysis, and drafted the manuscript. Y-mY and JZ helped design the study, collect the clinical data, and also revised this manuscript. L-tY and H-qT participated in designing and guiding the study to assure it run as intended. All authors contributed to the article and approved the submitted version.

## Conflict of Interest

The authors declare that the research was conducted in the absence of any commercial or financial relationships that could be construed as a potential conflict of interest.
