# Supplementary material for: Elevated Plasma Big Endothelin-1 at Admission Is Associated With Poor Short-Term Outcomes in Patients With Acute Decompensated Heart Failure
Source: Front Cardiovasc Med. 2021 Mar 11;8:629268. doi: 10.3389/fcvm.2021.629268 (PMC7990871; doi:10.3389/fcvm.2021.629268)
Supplement: Supplementary file 1 [file Data_Sheet_1.docx]

Supplementary data 1

The NRI and IDI results comparing predictive powers of big ET-1 and NT-proBNP

|  | Est. | Z-value | 95%CI | p value |
| --- | --- | --- | --- | --- |
| Difference in C statistic | 0.0324 |  | (-0.1032,0.0385) | 0.371 |
| IDI | 0.0253 | 1.799 | (-0.0023,0.0529) | 0.072 |
| NRI | 0.0540 | 0.485 | (-0.1636,0.2717) | 0.627 |

Supplementary data 2

Added discrimination and event-specific reclassification of big ET-1 levels to NT-proBNP

| IDI value | Standard error | Z-value for IDI | P value for IDI | IDI 95%CI | Relative IDI |
| --- | --- | --- | --- | --- | --- |
| 0.0185 | 0.0088 | 2.1123 | 0.0035 | (0.0013,0.0036) | 0.2822 |
| NRI value | Standard error | Z-value for NRI | P value for NRI | NRI 95%CI | %of events correctly reclassified |
| 0.5929 | 0.1086 | 5.3245 | <0.001 | (0.3799,0.8058) | 17% |

Supplementary data 3

Odds ratios of fixed covariates for primary endpoint

| Variables | OR | 95%CI | p value |
| --- | --- | --- | --- |
| **Big ET-1** | **1.520** | **1.133-2.039** | **0.005** |
| Age(yrs) | 0.980 | 0.955-1.004 | 0.106 |
| Gender | 0.927 | 0.431-1.991 | 0.845 |
| Lg NT-proBNP | 1.447 | 0.634-3.301 | 0.380 |
| SBP(mmHg) | 0.996 | 0.980-1.013 | 0.996 |
| LVEF(%) | 1.008 | 0.991-1.024 | 0.359 |
| Scr (μmol/L) | 0.194 | 0.025-1.499 | 0.116 |
| **WBC (×10^9^/L)** | **1.281** | **1.181-1.389** | **<0.001** |
| **TBIL (μmol/L)** | **1.017** | **1.006-1.029** | **0.004** |
